# What is the role of spatial processing in the decline of episodic memory in Alzheimer’s disease? The “mental frame syncing” hypothesis

**DOI:** 10.3389/fnagi.2014.00033

**Published:** 2014-03-10

**Authors:** Silvia Serino, Giuseppe Riva

**Affiliations:** ^1^Applied Technology for Neuro-Psychology Lab, IRCCS Istituto Auxologico ItalianoMilan, Italy; ^2^Department of Psychology, Università Cattolica del Sacro CuoreMilan, Italy

**Keywords:** episodic memory, spatial cognition, Alzheimer’s disease, allocentric frame of reference, egocentric frame of reference

## Abstract

The current theories on episodic memory suggest a crucial role of spatial processing for an effective retrieval. For a successful episodic recall, the long-term allocentric scene has to be translated into an egocentric scene. Here, we argue that a crucial role for an episodic retrieval is played by a “mental frame syncing” between two kinds of allocentric representations. This neurocognitive process allows an effective retrieval of our past experiences by synchronizing the allocentric view-point independent representation with the allocentric view-point dependent representation. If the “mental frame syncing” stops, even momentarily, it is difficult to reconstruct a coherent spatial scaffold upon which to effectively retrieve our previous events within an egocentric perspective. This is what apparently happens in Alzheimer’s disease: a break in the “mental frame syncing” between these two kinds of allocentric representations, underpinned by damage to the hippocampus, may contribute significantly to the early deficit in episodic memory.

## Introduction

In recent years, our understanding of the episodic memory system has undergone a radical revision, with a transition from a system directed exclusively to the past to a system oriented also to the future. Episodic memory has been historically defined as the ability to remember personal past events of a specific time and place–“what”, “where”, and “when”—and with a reference to themselves as participants of that events (Tulving, 1985, 2001, 2002).

On the basis of the most recent theories of episodic memory, several cognitive and neural processes work in parallel to support the “mental time travel” from past to present and future (for a review, see Roediger et al., 2007). On one side, an increasing number of studies has shown that when individuals remember the past or imagine the future, a comparable level of activation occurs in the regions of the brain comprising medial temporal and frontal lobes, posterior cingulate and retrosplenial cortex (RSC), and lateral parietal and temporal areas (Okuda et al., 2003; Addis et al., 2007, 2009a; Buckner and Carroll, 2007; Botzung et al., 2008; Spreng et al., 2009; Viard et al., 2011; Eichenbaum, 2013). On the other side, these converging empirical findings have led researchers to propose different theoretical ideas to emphasize the link between the retrieval of personal past events and the construction of future and imaginary events. In this direction, Schacter and Addis (2007a,b, 2009) proposed the constructive episodic simulation hypothesis, which highlights the role of memory in the construction of future events. This theoretical proposal highlights the adaptive function of the episodic memory system that extracts components of the past, then assembles and recombines them to remember the past and to imagine the future (Hardt et al., 2010; Howe, 2011; Schacter et al., 2011). Hassabis and Maguire (Hassabis et al., 2007a; Hassabis and Maguire, 2007, 2009) argued a critical role for a process of “scene construction” that links memory and imagination. This critical cognitive process is conceived to be more complex than “simple” visual imagery for individual objects (Kosslyn et al., 2001) because it relies on connecting several types of information (i.e., perceptual, semantic, contextual) into a coherent whole.

**Figure 1:**
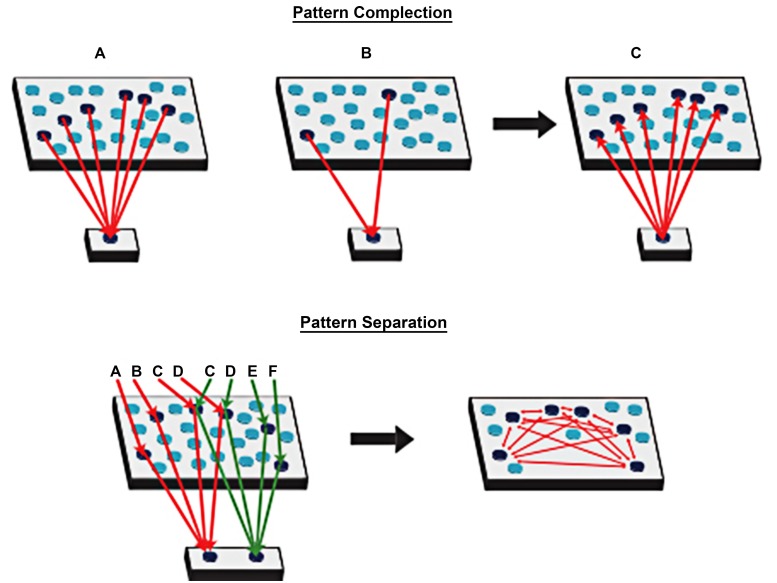
**Pattern completion: a set of neocortical patterns activated by a particular experience projects to the hippocampal formation and activates a unique set of synapses (the index)**. When there is a cue, output from the hippocampal formation projects back to the neocortex to activate the entire pattern. Pattern separation: the hippocampal formation supports pattern separation with the creation of different output firing pattern for similar inputs (Adapted from Rudy, 2009).

According to these new theories of episodic memory, it is crucial to understand the role of spatial processing in episodic memory. The theory behind spatial processing, the Cognitive Map Theory (O’Keefe and Nadel, 1978) was later advanced by the Multiple Trace Theory (Nadel and Moscovitch, 1997; Moscovitch and Nadel, 1998) and argued that a functional relationship between two kinds of memory, episodic memory and spatial memory, is under the control of the hippocampal complex. Nadel and Moscovitch later incorporated the episodic memory system into the Multiple Trace Theory (Nadel and Moscovitch, 1997; Moscovitch and Nadel, 1998). This theory states that the hippocampus provides a spatial framework linking all the neocortical representations related to each specific episode. Thanks to the hippocampus, individual are very able in storing and remembering episodes and retrieving them from pieces. A relevant approach of the mechanisms underlying the storing of episodic memories states the importance of solid and repeated associations between hippocampal sparse patterns of activity and distributed neocortical representations (Mcclelland et al., 1995). The origin of this approach is dated to Hirsh (1974) and Teyler and DiScenna (1986), who considered the hippocampus as a context-indexing device: on one side, the hippocampus supports pattern with the creation of different output firing pattern for similar inputs; on the other side, the memory of an episode is retrieved by reactivating an hippocampal pointer to it thanks to pattern completion (see Figure 1).

According to these theories, an allocentric spatial representation (namely, a representation coding object-to-object relations, independent of an individual’s orientation) is stored in the hippocampus (O’Keefe and Nadel, 1978). This hypothesis came from the pioneering discovery of “place cells” in the hippocampus of freely moving rats (O’Keefe and Dostrovsky, 1971) and recently received strong support by studies involving primates (Ono et al., 1993) and humans (Ekstrom et al., 2003). Specifically, within the hippocampus, the region CA3, receiving inputs from the entorhinal cortex, forms an allocentric representation of the scene toward which the animal orients, whereas the neurons in CA1, receiving inputs from CA3 via Schaffer’s collaterals, rapidly encodes allocentric representations involving abstract object-to-object information (Robertson et al., 1998; Rolls, 2007). In this prospective, it is possible to distinguish between allocentric representation formed by CA3 (*allocentric view-point dependent representation*) and allocentric representation formed by CA1 (*allocentric view-point independent representation*) (Behrendt, 2013). Recently, Burgess and colleagues proposed a model that addresses the function of spatial processing in episodic retrieval (Burgess et al., 2001; Byrne et al., 2007). Based on the reciprocal connectivity between the hippocampus and neocortical regions, their Boundary Vector Cells Model provides support for a crucial role of the hippocampal place cells in retrieving a spatially coherent scene. When prompted by a retrieval cue, the full spatial representation of the environment can be retrieved through the process of pattern completion (Byrne et al., 2007). Though allocentric (specifying scene elements in terms of north, south east, west), this representation is translated to an egocentric representation (specifying scene elements in terms of left, right, ahead of the individual) thanks to information from other cells. The cells that contribute to this representation are the hippocampal place cells that inform the viewpoint location (Ekstrom et al., 2003), the head-direction cells orient the viewing direction (Taube, 1998), and the grid cells interpret the self-motion signals (Hafting et al., 2005). The RSC transforms the long-term allocentric representations into egocentric ones using head information to compensate for the rotation offset between different spatial coordinates (Maguire, 2001; Vann et al., 2009).

Starting from Burgess and colleagues’ model, we argue that it may be critical to include another neurocognitive process to the episodic retrieval. As stated, episodic recall (including the simulation of future or imaginary events) may be traceable to retrieval cues (encoded in neocortical areas) inducing the reinstatement of neuronal assemblies in CA3 via direct entorhinal input and in CA1 via Schaffer’s collaterals.

We suggest that for an effective episodic retrieval it is crucial that allocentric view-point independent representation has to be synced with the allocentric view-point dependent representation. In Alzheimer’s disease (AD) a break in the “mental frame syncing” between these two allocentric representations occurs, caused by damage to the hippocampus, and it may contribute significantly to the early impairment of episodic memory.

## The role of spatial processing in episodic memory: the “mental frame syncing” hypothesis

The retrieval of episodic memories is widely recognized to be a reconstructive process as opposed to a straightforward retrieval of a memory (Bartlett, 1932; Hassabis and Maguire, 2007; Schacter and Addis, 2007a, 2009). Parallel processes work together to create a flexible and highly adaptive system to support the self-projection between past and future (Buckner and Carroll, 2007). As noted above, the hippocampus plays a critical role in retrieving past experience, imagining experiences and envisioning the future by a spatial coherent framework. However, what is the specific role of spatial processing in episodic memory?

In other terms, as explained in the Boundary Vector Cells Model (Byrne et al., 2007), for an effective episodic retrieval the long-term allocentric representation (independent of individual’s orientation) has to be translated into an egocentric representation (dependent of individual’s orientation).

However, like when we orient ourselves in an environment, even when we evoke personal memories of our past, first we have to identify relevant “objects” in real and/or cognitive space, and we remember their positions with respect to another (allocentric view-point independent representation). Second, we have to identify our current position and a specific viewpoint on this allocentric map (allocentric view-point dependent representation). To do so, we have to synchronize the allocentric view-point independent representation with allocentric view-point dependent representation (namely, the “mental frame syncing”).

Following this perspective, the “mental frame syncing” may be defined as a cognitive process that allows an effective retrieval of our past experiences by synchronizing the view-point independent representation with the allocentric view-point dependent representation.

This process permits the translation from the allocentric representation into the egocentric one.

In AD a break in the “mental frame syncing” may significantly contribute to the early deficit in the episodic memory. In support of this hypothesis, we present a brief review of neurocognitive evidence. In the early stages of AD, indeed, brain regions that are primarily affected are those involved in the neural circuit that is presumed to be critical for both spatial and episodic memory system. This may lead to investigate which is the crucial cognitive process that could be the link between this two system.

## A break in “the mental frame syncing”: some evidence in Alzheimer’s disease

The earliest neurofibrillary tangles of AD-related neuropathologic changes usually begin in the medial temporal lobe and related structures, especially the hippocampus (Braak and Braak, 1991, 1996; Braak et al., 2006; Alafuzoff et al., 2008). Recent evidence demonstrated that the amyloid plaques, the other major component of AD neuropathologic change, occur long before the onset of clinical symptoms (Morris et al., 1996; Dickson, 1997; Thal et al., 2002). The initial amyloid plaques tend to have a higher density in a set of strongly interconnected cortical areas (e.g., the posterior cingulate cortex, the inferior parietal lobule, and the medial prefrontal cortex) that project into the medial temporal lobes. One of the most crucial results of neurodegenerative process in AD is cerebral atrophy, which can be particularly detected in temporal areas such as the hippocampus (Jack et al., 2004) and enthorinal cortex (Du et al., 2003). An increasing number of studies showed longitudinal hippocampal change (Fox et al., 1996; Jack et al., 2000; Kaye et al., 2005), including a recent meta-analysis that showed the overall hippocampal atrophy rate is 4.6% in AD subjects (Barnes et al., 2009). Some recent studies have tried to investigate which specific hippocampal areas would manifest neuronal dysfunction in the earliest stage of the disease. Padurariu et al. (2012) have recently showed that decrease of hippocampal neuronal density is more prominent especially in the CA1 and CA3 hippocampal areas. As noted earlier, all these early neurodegenerative processes significantly impair the neural network that is presumed to be critical for both spatial and episodic memory system.

Specifically, tests of spatial cognition could be useful to detect early deficits in AD. Some recent fMRI studies have focused on the hyperactivity of specific region of hippocampus in AD. Miller et al. (2008) tested 25 older individuals with Mild Cognitive Impairment (MCI) in performing a visual scene encoding task during fMRI scanning. Then, participants are followed clinically for at least 4 years after scanning. Findings confirmed higher activation in the hippocampus, and showed that it this was predictive of subsequent cognitive decline and conversion to AD. Indeed, patients with MCI were progressing more frequently to AD compared to healthy age-matched controls (for a review, see Mitchell and Shiri-Feshki, 2009).

**Figure 2:**
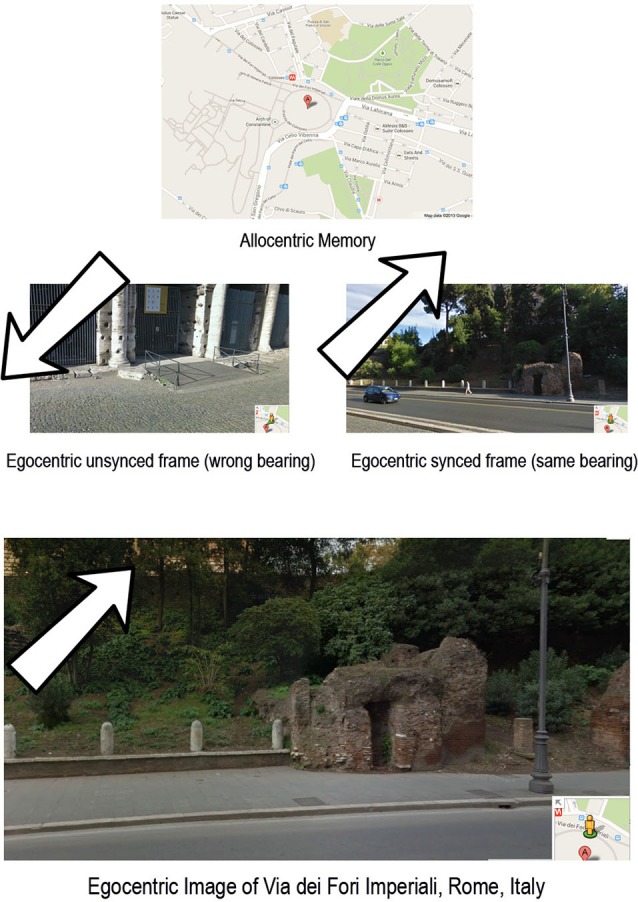
**The “mental frame syncing” may be defined as a neurocognitive process that allows an effective retrieval of our past experiences by synchronizing the view-point independent representation with the allocentric view-point dependent representation**. If the mental frame syncing stops, the reconstructed scenario is useless because the view-point is not similar to those that had activated the episodic retrieval.

Furthermore, Yassa et al. (2010) found hyperactive BOLD signals in the CA3 areas during a continuous recognition task designed to emphasize pattern separation in patients with amnestic MCI. In short, these results suggest that the test evaluating the spatial cognition and, especially, the pattern separation can be used to monitor the clinical progression from MCI to AD. This “inflexibility” in spatial abilities may lead to an impoverished allocentric view-point dependent representation. This evidence gives support to the hypothesis that in the early stages of AD, brain regions that are primarily affected are those involved in the neural circuit that supports the processing of spatial representations and their mutual relations. On one hand, damages in specific areas of hippocampus the ability in constructing and storing a long-term allocentric representation. Specifically, the hippocampal neurodegeneration may seriously influence the reinstatement of neuronal assemblies in CA3: an impoverished allocentric view-point dependent representation could result from that process (see Figure 2). So, based on this evidence, early damage in the hippocampus may provoke a break in the “mental frame syncing”: An allocentric view-point independent representation may not be effectively synced with an allocentric view-point dependent representation (Serino and Riva, 2013). Furthermore, this may explain the co-occurrence of episodic and spatial deficits in the early stages of AD. When there is a break in the “mental frame syncing”, as we suppose occurring in AD, the retrieved representation may present fewer episodic event-specific details (Addis et al., 2009b; Gamboz et al., 2010) and lack spatial coherence (Hassabis et al., 2007b). Indeed, Gamboz et al. (2010) tested 14 patients with amnesic Mild Cognitive Impairment (aMCI) and 14 age-matched controls in mentally re-experiencing and pre-experiencing autobiographical episodes. Results showed that aMCI patients produced fewer episodic event-specific details in their recollections with simulation of past and future events, as compared to controls. Furthermore, Addis et al. (2009b) tested the cognitive ability of sixteen mild AD patients and sixteen age-matched controls in producing past and future events using an adapted version of the Autobiographical Interview. AD patients showed deficits in both remembering past events and simulating future events, generating fewer internal and external episodic elements than healthy controls. The presence of episodic event-specific details concerns the synchronization with the allocentric view-point representation, whereas the spatial coherence is allowed by the allocentric view-point representation.

## Conclusion and future directions

In the last 30 years, neuropsychological research has tried to identify the most salient and earliest clinical symptoms characterizing the dementia associated with AD (Flicker et al., 1984; Morris et al., 1989; Welsh et al., 1991, 1992; Locascio et al., 1995; Albert, 1996, 2011; Weintraub et al., 2012). In addition to episodic memory impairment, a decline in spatial memory is one of the earliest clinical manifestations of AD (Henderson et al., 1989; Passini et al., 1995; Cherrier et al., 2001; Monacelli et al., 2003; Guariglia and Nitrini, 2009; Laczó et al., 2011; Gazova et al., 2012; Lithfous et al., 2013).

Based on this neurocognitive evidence, a deficit in the “mental frame syncing” between the allocentric view-point dependent and the allocentric view-point independent representation may contribute to early manifestations of episodic memory impairment in AD. The current theories of episodic memory help us to understand the malleability of the human memory, and specifically the reconstructive nature of episodic retrieval. When we remember an event, our mind is engaged in a process of reconstructing a scenario. Recent neuroimaging and cognitive studies suggest that the same thing happens when we envision our future and/or we create imaginary events. We extract pieces of information from our past and recombine them in a creative and flexible manner according to our needs. This involves a set of brain regions comprising medial temporal and frontal lobes, posterior cingulate and RSC, and lateral parietal and temporal areas, which highlights the key role of medial temporal lobe structures, particularly the hippocampus. The hippocampus plays a central role in episodic memory, but also has a major role in spatial cognition. According to the most recent theories, the hippocampus provides spatial scaffold upon which to build a scenario. Therefore, it is crucial to consider the specific role of spatial processing in episodic memory. Starting from Burgess and colleagues’ model (2001), we argue that it may be crucial to include another cognitive process to the egocentric-allocentric translation system. It is crucial that the allocentric view-point dependent are synced with allocentric view-point independent representation: The result of this “mental frame syncing” permits an effectively retrieval of our past experiences.

In AD a break in the “mental frame syncing” between these two kinds of allocentric representations may contribute significantly to the early deficit in episodic memory. In the early stages of AD, brain regions that are primarily affected are those involved in the neural circuit that supports that supports the processing of allocentric and their mutual relations. On the other hand, spatial deficits occurring in first stages of disease underline how allocentric representations are early impaired, and affect the episodic retrieval. We suggest that a cognitive impairment in the “mental frame syncing”, underpinned by damage to the hippocampus, may become a crucial cognitive marker both for early and differential diagnosis of AD. Identification of these cognitive deficits could facilitate the development of early rehabilitation interventions and the possibility to identify individuals most at risk of progression to AD during the preclinical stages of the disease (Sperling et al., 2011). Indeed, the cognitive assessment continues to provide reliable cognitive markers of AD that are crucial both for early and differential diagnosis. Furthermore, for neurorehabiliation interventions, a recent study showed that older individuals who use flexible spatial navigational strategies in their everyday lives may have increased gray matter in the hippocampus, and it enhances their probability of healthy and successful aging (Konishi and Bohbot, 2013). This “flexible spatial strategies” emphasizing the abilities orientating oneself in relation to different landmarks, in opposition to the “response strategy” that involves learning a series of stimulus-response associations, ìmay underline a good “mental frame syncing” between the view-point independent representation and the allocentric view-point dependent representation.

In conclusion, as for any new hypothesis, more research is needed before it can be retained or discarded. In particular, new research studies are required to further explore the link between the “mental frame syncing” and the episodic retrieval in AD.

## Author contributions

Silvia Serino developed the first draft manuscript into the final version suitable for publication. Giuseppe Riva supervised the rationale in its scientific design. All authors read and approved the final manuscript.

## Conflict of interest statement

The authors declare that the research was conducted in the absence of any commercial or financial relationships that could be construed as a potential conflict of interest.
